# Supplementary material for: Patterns of Intron Gain and Loss in Fungi
Source: PLoS Biol. 2004 Nov 30;2(12):e422. doi: 10.1371/journal.pbio.0020422 (PMC532390; doi:10.1371/journal.pbio.0020422)
Supplement: Table S1 — Also available at http://genes.mit.edu/NielsenEtAl/. (4.3 MB ZIP). [file pbio.0020422.st001.zip › NielsenEtAl/html/419.html]

AN7194.1.NCU01419.1.MG00738.1.FG10124.1


```
 CLUSTAL W (1.82) Multiple Sequence Alignments - Introns Inserted


Sequence 1: MG00738.1	343 aa
Sequence 2: FG10124.1	335 aa
Sequence 3: NCU01419.1	338 aa
Sequence 4: AN7194.1	330 aa
Alignment Length: 347 aa
Number Identitical Residues: 152 aa
Alignment Score (without introns) 7959


MG00738.1 	-MPETMKAVD1VRGGAGPASALFINAETPRPTPGAGQVLVKVSAFGLNRMDLLQREGKYP
NCU01419.1	MAPKTMKAVD1IKNGKGSADNLFINPDTPLPQAQPGQALVKVKAFGLNRMDLIQREGHYP
FG10124.1 	-MTDYIKAVD1IRGGTGERDALFINDKTPKPSAAEGQAIVKIKAFGINRMDIIQRRGFYP
AN7194.1  	-----MRAVA1IKGGKGPADALYIDN-IPVPEYGPSQALVKVKAFGLNRMDLLQREGQYP
          	     ::**  ::.* *  . *:*:   * *    .*.:**:.***:****::**.* **

MG00738.1 	LPPQAPKTLGVEFSGVIESLGDFSASASTDADGIPFKKGDAVFGLAYGDAYAEYIAVSAR
NCU01419.1	LPPQAPSTLGVEFSGTIESFG--------DGDHGDFNVGDEVFGLAYGGAYAEYIAVSSK
FG10124.1 	LPPQAPKTLGVEFSGIIESFG--------PGDHGAFTEGDEVFGLAYGGAYAEYIAVSTR
AN7194.1  	VPPQAPATLGVEFSGTIAELG--------DGATEDFKVGDEVFGLAYGGAYAEYIAVATG
          	:***** ******** * .:*         .    *. ** *******.********:: 

MG00738.1 	MLMHKPAGLTHEQAAGIPE~TFITATQALHLVGG--FAKGDRVLWHAGASGVSIAGAQLA
NCU01419.1	MLLHKPAHLSFEQAAGVPE~TWITATQALHFVLGGIGEQGKTILWHAGASGVSIAGIQLS
FG10124.1 	MLLHKPSSIDFTTCAGVPE~AWITATQALHLVLG--FTKGKSILWHAGASGVSVAGIQLS
AN7194.1  	MLIHKPKELSWEEAAGIPE0TWITATQALHLVGA--FKPGNSVLWHAGASSVSIAGIQLA
          	**:***  :    .**:** ::********:* .     *. :*******.**:** **:

MG00738.1 	RRAGAGAVFSTAGSDDKCAFLREELGVDGAFNYKTQDWAEEVRRATGGRGVDLIVDFVGA
NCU01419.1	KLAGAKQIFATAGSADKCKFIENELGATAAFNYKEQDWVEEIMKKTDGRGVDLVVDFIGA
FG10124.1 	RVAGASAVYATAGSQDKLDFITSKLGATAAFNYKTQDWAEEIKKATGGKGVDYIVDFVGG
AN7194.1  	KAAGASAIYVTAGSDEKISFCVNQLGATAGFNYRTQNWAEELSKATNGRGVDVIVDYIGA
          	: ***  :: **** :*  *  .:**. ..***: *:*.**: : *.*:*** :**::*.

MG00738.1 	DYFSKNMDAAAQEGRIVCLGLLSGAVVKEVNVAQLLFKRLRIEGSTLRARDEEYQGMLRD
NCU01419.1	SYFQKNLDVVARDGRICMLGLMGGAVADKVDISKLLYKRARVEGSTLRSRDENYQGQLRD
FG10124.1 	NYFQKNLDVAARDGRMVLLGTLSGAKVPDADISQILFKRLRIEGSTLRSRDEEYQGELRD
AN7194.1  	GYFQDNLKSAALDGRIVNLAFLGGIKVENVDISYFLRKRIRYEGSTLRSRDEEYQRKLRN
          	.**..*:. .* :**:  *. :.*  . ..::: :* ** * ******:***:**  **:

MG00738.1 	RLEE-CLPQFETGELKVFVDKVLSWEQIQEAHELLEKNVTRGKIICTIP
NCU01419.1	KLSE-YLPKFESGELKIFIDTVLPFEEIVKAHKQLEANTTMGKIICTVS
FG10124.1 	RLEK-YIPEFESGNLKIFVDEVFPWEQIQEAHKHMEDAKNLGKIICTIS
AN7194.1  	MLVDNALEKLRNKEFKVFVEKVFKFDDVVEAHKLMESNQTKGKIICTI-
          	 * .. : ::.. ::*:*:: *: :::: :**: :*   . ******:
```
